# Addendum: Šarić-Kundalić, B.; Fialová, S.; Dobeš, C.; Ölzant, S.; Tekeľová, D.; Grančai, D.; Reznicek, G.; Saukel, J. Multivariate Numerical Taxonomy of *Mentha* Species, Hybrids, Varieties and Cultivars. *Sci. Pharm.* 2009, *77*, 851–876

**DOI:** 10.3390/scipharm84040752

**Published:** 2016-12-02

**Authors:** Broza Šarić-Kundalić, Silvia Fialová, Christoph Dobeš, Silvester Ölzant, Daniela Tekeľová, Daniel Grančai, Gottfried Reznicek, Johannes Saukel

**Affiliations:** 1 Department of Pharmacognosy, University of Vienna, Althanstraße 14, 1090 Vienna, Austria; christoph.dobes@bfw.gv.at (C.D.); silvester.oelzant@ages.at (S.Ö.); gottfried.reznicek@univie.ac.at (G.R.); johannes.saukel@univie.ac.at (J.S.); 2 Department of Pharmacognosy and Botany, Faculty of Pharmacy, Comenius University, Odbojarov 10, 83 232 Bratislava, Slovakia; tekelova@fpharm.uniba.sk (D.T.); grancai@fpharm.uniba.sk (D.G.)

In the published paper [1], the following plant species are listed: *Mentha longifolia* var. *lavanduliodora*, *Mentha spicata* and *Mentha spicata* var. *crispa*. We have been informed by Szkukalek Attila that these samples had been grown in his private garden in Komarno, Slovakia. This information should have been included in the acknowledgment section of the manuscript. We apologize for any inconvenience.
